# Supplementary material for: Modeling glioblastoma heterogeneity as a dynamic network of cell states
Source: Mol Syst Biol. 2021 Sep 16;17(9):e10105. doi: 10.15252/msb.202010105 (PMC8444284; doi:10.15252/msb.202010105)
Supplement: Supplementary file 6 — Source Data for Figure 5 [file MSB-17-e10105-s004.zip › Figure5A_sourcedata/GSEA_3017/nosigdb_stateF.GseaPreranked.1622795015644/gsea_report_for_na_neg_1622795015644.html]

Report for na\_neg 1622795015644 [GSEA]

| GS  follow link to MSigDB | GS DETAILS | SIZE | ES | NES | NOM p-val | FDR q-val | FWER p-val | RANK AT MAX | LEADING EDGE || 1 | DOROSZKO\_U3013MG\_CLUSTER\_2 | Details ... | 178 | -0.86 | -6.16 | 0.000 | 0.000 | 0.000 | 265 | tags=83%, list=12%, signal=87% |
| 2 | DOROSZKO\_U3180MG\_CLUSTER\_3 | Details ... | 286 | -0.78 | -6.13 | 0.000 | 0.000 | 0.000 | 419 | tags=76%, list=19%, signal=82% |
| 3 | DOROSZKO\_U3054MG\_CLUSTER\_4 | Details ... | 328 | -0.76 | -6.07 | 0.000 | 0.000 | 0.000 | 581 | tags=83%, list=26%, signal=95% |
| 4 | RICHARDS\_NOWAKOWSKI\_SCIENCE\_2017\_IPC\_DIV2\_UPREG | Details ... | 431 | -0.72 | -6.04 | 0.000 | 0.000 | 0.000 | 590 | tags=77%, list=26%, signal=84% |
| 5 | RICHARDS\_NOWAKOWSKI\_SCIENCE\_2017\_MGE\_DIV\_UPREG | Details ... | 336 | -0.75 | -5.97 | 0.000 | 0.000 | 0.000 | 558 | tags=80%, list=25%, signal=91% |
| 6 | RICHARDS\_NOWAKOWSKI\_SCIENCE\_2017\_IPC\_DIV1\_UPREG | Details ... | 336 | -0.73 | -5.81 | 0.000 | 0.000 | 0.000 | 465 | tags=72%, list=21%, signal=77% |
| 7 | FELDMAN\_2020\_G1 | Details ... | 133 | -0.85 | -5.81 | 0.000 | 0.000 | 0.000 | 323 | tags=89%, list=14%, signal=97% |
| 8 | RICHARDS\_NOWAKOWSKI\_SCIENCE\_2017\_MGE\_IPC2\_UPREG | Details ... | 298 | -0.75 | -5.80 | 0.000 | 0.000 | 0.000 | 416 | tags=71%, list=18%, signal=76% |
| 9 | GAROFANO\_IAVARONE\_PPR\_SUBTYPE | Details ... | 243 | -0.76 | -5.79 | 0.000 | 0.000 | 0.000 | 429 | tags=74%, list=19%, signal=82% |
| 10 | LARSSON\_STATE\_4 | Details ... | 96 | -0.89 | -5.69 | 0.000 | 0.000 | 0.000 | 192 | tags=86%, list=9%, signal=90% |
| 11 | RICHARDS\_NOWAKOWSKI\_SCIENCE\_2017\_MGE\_IPC1\_UPREG | Details ... | 302 | -0.69 | -5.39 | 0.000 | 0.000 | 0.000 | 432 | tags=66%, list=19%, signal=70% |
| 12 | RICHARDS\_ZHONG\_NATURE\_2018\_NPCS\_UPREG | Details ... | 102 | -0.83 | -5.29 | 0.000 | 0.000 | 0.000 | 262 | tags=80%, list=12%, signal=87% |
| 13 | FELDMAN\_2020\_S-G2 | Details ... | 242 | -0.69 | -5.27 | 0.000 | 0.000 | 0.000 | 332 | tags=67%, list=15%, signal=71% |
| 14 | NB\_KI\_CLUSTER5 | Details ... | 78 | -0.84 | -5.17 | 0.000 | 0.000 | 0.000 | 331 | tags=91%, list=15%, signal=103% |
| 15 | FELDMAN\_2020\_G2-M | Details ... | 224 | -0.67 | -5.05 | 0.000 | 0.000 | 0.000 | 380 | tags=70%, list=17%, signal=75% |
| 16 | SEURAT\_CELLCYCLE | Details ... | 74 | -0.81 | -4.81 | 0.000 | 0.000 | 0.000 | 337 | tags=84%, list=15%, signal=95% |
| 17 | RICHARDS\_NOWAKOWSKI\_SCIENCE\_2017\_RG\_EARLY\_UPREG | Details ... | 211 | -0.59 | -4.44 | 0.000 | 0.000 | 0.000 | 304 | tags=50%, list=13%, signal=52% |
| 18 | RICHARDS\_NEFTEL\_CELL\_2019\_G2\_M | Details ... | 40 | -0.85 | -4.41 | 0.000 | 0.000 | 0.000 | 258 | tags=90%, list=11%, signal=100% |
| 19 | SUVA\_G2M\_PMID\_31327527 | Details ... | 40 | -0.85 | -4.30 | 0.000 | 0.000 | 0.000 | 258 | tags=90%, list=11%, signal=100% |
| 20 | FELDMAN\_2020\_S | Details ... | 108 | -0.63 | -4.22 | 0.000 | 0.000 | 0.000 | 337 | tags=68%, list=15%, signal=76% |
| 21 | DALMO\_BARCODENUMBER\_UNPUB |  | 168 | -0.57 | -4.08 | 0.000 | 0.000 | 0.000 | 214 | tags=53%, list=9%, signal=54% |
| 22 | RICHARDS\_NOWAKOWSKI\_SCIENCE\_2017\_MGE\_RG2\_UPREG |  | 203 | -0.55 | -4.05 | 0.000 | 0.000 | 0.000 | 593 | tags=66%, list=26%, signal=81% |
| 23 | DOROSZKO\_U3054MG\_CLUSTER\_6 |  | 61 | -0.68 | -3.89 | 0.000 | 0.000 | 0.000 | 671 | tags=93%, list=30%, signal=129% |
| 24 | DOROSZKO\_U3180MG\_CLUSTER\_4 |  | 94 | -0.61 | -3.84 | 0.000 | 0.000 | 0.000 | 682 | tags=85%, list=30%, signal=117% |
| 25 | IVY\_CT |  | 117 | -0.57 | -3.83 | 0.000 | 0.000 | 0.000 | 214 | tags=45%, list=9%, signal=47% |
| 26 | FELDMAN\_2020\_NEURAL G0 |  | 230 | -0.50 | -3.78 | 0.000 | 0.000 | 0.000 | 323 | tags=47%, list=14%, signal=49% |
| 27 | GAROFANO\_IAVARONE\_LIST\_OF\_NEURAL\_PROGENITOR\_MARKER\_GENES |  | 56 | -0.67 | -3.74 | 0.000 | 0.000 | 0.000 | 267 | tags=63%, list=12%, signal=69% |
| 28 | FELDMAN\_2020\_LATEG1 |  | 57 | -0.67 | -3.67 | 0.000 | 0.000 | 0.000 | 214 | tags=56%, list=9%, signal=60% |
| 29 | RICHARDS\_NEFTEL\_CELL\_2019\_G1\_S |  | 22 | -0.80 | -3.40 | 0.000 | 0.000 | 0.000 | 419 | tags=95%, list=19%, signal=116% |
| 30 | SUVA\_G1S\_PMID\_31327527 |  | 22 | -0.80 | -3.39 | 0.000 | 0.000 | 0.000 | 419 | tags=95%, list=19%, signal=116% |
| 31 | FELDMAN\_2020\_M-EARLYG1 |  | 80 | -0.55 | -3.38 | 0.000 | 0.000 | 0.000 | 215 | tags=50%, list=10%, signal=53% |
| 32 | RICHARDS\_CAHOY\_JNEUROSCI\_2010\_ASTRO\_YOUNG |  | 286 | -0.44 | -3.38 | 0.000 | 0.000 | 0.000 | 369 | tags=41%, list=16%, signal=43% |
| 33 | PARADA\_T1\_UP\_26461091 |  | 41 | -0.56 | -2.92 | 0.000 | 0.000 | 0.000 | 249 | tags=51%, list=11%, signal=57% |
| 34 | NB\_KI\_CLUSTER3 |  | 49 | -0.52 | -2.81 | 0.000 | 0.000 | 0.000 | 813 | tags=86%, list=36%, signal=131% |
| 35 | PARADA\_T2\_UP\_26461091 |  | 33 | -0.55 | -2.73 | 0.000 | 0.000 | 0.000 | 249 | tags=45%, list=11%, signal=50% |
| 36 | RICHARDS\_CAHOY\_JNEUROSCI\_2016\_CULTURED\_ASTROGLIA |  | 381 | -0.32 | -2.60 | 0.000 | 0.000 | 0.000 | 252 | tags=25%, list=11%, signal=24% |
| 37 | SINGH\_SOX2\_UPREGULATED\_TRANSCRIPTS\_PMID\_28122245 |  | 145 | -0.37 | -2.53 | 0.000 | 0.000 | 0.000 | 182 | tags=22%, list=8%, signal=22% |
| 38 | RICHARDS\_NOWAKOWSKI\_SCIENCE\_2017\_MGE\_IPC3\_UPREG |  | 50 | -0.44 | -2.38 | 0.000 | 0.000 | 0.004 | 381 | tags=40%, list=17%, signal=47% |
| 39 | OLSEN\_ADRENERGIC |  | 32 | -0.45 | -2.16 | 0.002 | 0.000 | 0.022 | 377 | tags=41%, list=17%, signal=48% |
| 40 | VANGRONINGEN\_ADRN |  | 117 | -0.32 | -2.13 | 0.000 | 0.000 | 0.027 | 368 | tags=31%, list=16%, signal=35% |
| 41 | FELDMAN\_2020\_LOWRNA |  | 378 | -0.26 | -2.12 | 0.000 | 0.000 | 0.030 | 507 | tags=35%, list=22%, signal=38% |
| 42 | LARSSON\_STATE\_1 |  | 39 | -0.34 | -1.65 | 0.021 | 0.021 | 0.715 | 537 | tags=49%, list=24%, signal=63% |
| 43 | LARSSON\_STATE\_3 |  | 39 | -0.30 | -1.52 | 0.051 | 0.044 | 0.939 | 175 | tags=21%, list=8%, signal=22% |
| 44 | KLING\_GBM\_PHENO\_PMID\_27667176 |  | 48 | -0.28 | -1.51 | 0.047 | 0.047 | 0.954 | 132 | tags=19%, list=6%, signal=19% |
| 45 | ALMSTEDT\_RISK |  | 16 | -0.38 | -1.46 | 0.091 | 0.061 | 0.973 | 127 | tags=25%, list=6%, signal=26% |
| 46 | RICHARDS\_NOWAKOWSKI\_SCIENCE\_2017\_U3\_UPREG |  | 192 | -0.19 | -1.44 | 0.027 | 0.065 | 0.981 | 441 | tags=29%, list=20%, signal=33% |
| 47 | TF\_CENSUS\_TAIPALE |  | 164 | -0.15 | -1.07 | 0.321 | 0.376 | 1.000 | 103 | tags=7%, list=5%, signal=7% |
| 48 | ALMSTEDT\_17Q\_MSIGDB |  | 81 | -0.16 | -0.98 | 0.503 | 0.528 | 1.000 | 149 | tags=11%, list=7%, signal=11% |
| 49 | NB\_KI\_CLUSTER24 |  | 20 | -0.21 | -0.86 | 0.612 | 0.727 | 1.000 | 1790 | tags=100%, list=79%, signal=481% |
| 50 | NB\_KI\_CLUSTER7 |  | 25 | -0.13 | -0.57 | 0.931 | 0.988 | 1.000 | 372 | tags=24%, list=17%, signal=28% |
| 51 | RICHARDS\_NOWAKOWSKI\_SCIENCE\_2017\_NEN\_EARLY1\_UPREG |  | 177 | -0.06 | -0.45 | 1.000 | 0.994 | 1.000 | 514 | tags=25%, list=23%, signal=30% |
Table: Gene sets enriched in phenotype **na **[plain text format]****
